# Supplementary material for: Influence of Sociodemographic and Lifestyle Factors on Depression and Anxiety in Patients with Rheumatoid Arthritis in Saudi Arabia
Source: Int J Environ Res Public Health. 2025 Oct 25;22(11):1625. doi: 10.3390/ijerph22111625 (PMC12652553; doi:10.3390/ijerph22111625)
Supplement: Supplementary file 1 [file ijerph-22-01625-s001.zip › ijerph-3851490-supplementary.pdf]

# Supplymentary Material S1

# Hospital Anxiety and Depression Scale (HADS)

Please put this (√) in front of the appropriate answer

**Tick the box beside the reply that is closest to how you have been feeling in the past week.**
**Don’t take too long over you replies: your immediate is best.**

| D | A | | D | A | |
|---|---|---|---|---|---|
| | | **I feel tense or 'wound up':** | | | **I feel as if I am slowed down:** |
| | 3 | Most of the time | 3 | | Nearly all the time |
| | 2 | A lot of the time | 2 | | Very often |
| | 1 | From time to time, occasionally | 1 | | Sometimes |
| | 0 | Not at all | 0 | | Not at all |
| | | | | | |
| | | **I still enjoy the things I used to enjoy:** | | | **I get a sort of frightened feeling like 'butterflies' in the stomach:** |
| 0 | | Definitely as much | | 0 | Not at all |
| 1 | | Not quite so much | | 1 | Occasionally |
| 2 | | Only a little | | 2 | Quite Often |
| 3 | | Hardly at all | | 3 | Very Often |
| | | | | | |
| | | **I get a sort of frightened feeling as if something awful is about to happen:** | | | **I have lost interest in my appearance:** |
| | 3 | Very definitely and quite badly | 3 | | Definitely |
| | 2 | Yes, but not too badly | 2 | | I don't take as much care as I should |
| | 1 | A little, but it doesn't worry me | 1 | | I may not take quite as much care |
| | 0 | Not at all | 0 | | I take just as much care as ever |
| | | | | | |
| | | **I can laugh and see the funny side of things:** | | | **I feel restless as I have to be on the move:** |
| 0 | | As much as I always could | | 3 | Very much indeed |
| 1 | | Not quite so much now | | 2 | Quite a lot |
| 2 | | Definitely not so much now | | 1 | Not very much |
| 3 | | Not at all | | 0 | Not at all |
| | | **Worrying thoughts go through my mind:** | | | **I look forward with enjoyment to things:** |
| | 3 | A great deal of the time | 0 | | As much as I ever did |
| | 2 | A lot of the time | 1 | | Rather less than I used to |
| | 1 | From time to time, but not too often | 2 | | Definitely less than I used to |
| | 0 | Only occasionally | 3 | | Hardly at all |
| | | | | | |
| | | **I feel cheerful:** | | | **I get sudden feelings of panic:** |
| 3 | | Not at all | | 3 | Very often indeed |
| 2 | | Not often | | 2 | Quite often |
| 1 | | Sometimes | | 1 | Not very often |
| 0 | | Most of the time | | 0 | Not at all |
| | | | | | |
| | | **I can sit at ease and feel relaxed:** | | | **I can enjoy a good book or radio or TV** |

| | | | | | | |
|---|---|---|---|---|---|---|
| | | | | | | **program:** |
| | 0 | | Definitely | 0 | | Often |
| | 1 | | Usually | 1 | | Sometimes |
| | 2 | | Not Often | 2 | | Not often |
| | 3 | | Not at all | 3 | | Very seldom |

Please check you have answered all the questions

Scoring:

Total score: Depression (D) ___________ Anxiety (A) ______________

0-7 = Normal

8-10 = Borderline abnormal (borderline case)

11-21 = Abnormal (case)

# Hospital Anxiety Depression Scale (HADS

هذا الاستبيان يساعد الطبيب لمعرفة مشاعرك وقراءة أحاسيسك ,لذا يرجى إحاطة الرقم الموازي لأفضل اختيار يصف حالتك خلال الأسبوع الماضي.ليس من المطلوب الاسغراق في التفكير لإختيار الإجابة ,وإنما تفضل الإجابات العفوية التلقائية.

يرجى وضع علامة صح (√) أمام الإجابة المناسبة

| | |
|---|---|
| 1.أحس بأني هامد (فاقد للطاقة):<br>D<br>☐ تقريبا في كل وقت<br>☐ في كثير من الأحيان<br>☐ في بعض الأوقات<br>☐ لا أشعر بذلك مطلقاً | 2.أشعر بالتوتر الشديد:<br>A<br>☐ أكثر الوقت<br>☐ عدة مرات<br>☐ أحياناً<br>☐ لا أشعر بذلك مطلقاً |
| 3.ينتابني شعور بالخوف:<br>A<br>☐ لا,على الإطلاق<br>☐ أحياناً<br>☐ كثيراً<br>☐ في أغلب الأوقات | 4.أنا لا زلت أتمتع بالأشياء التي اعتدت أن أستمتع بها :<br>D<br>☐ بالتأكيد,كما كنت<br>☐ ليس تماماً<br>☐ قليلاً<br>☐ بالكاد,على الإطلاق |
| 5.لقد فقدت الاهتمام بمظهري:<br>D<br>☐ بالتأكيد فقدت كل الاهتمام<br>☐ أنا لا أهتم بمظهري كما يجب أن أهتم<br>☐ قد لا أعتني بمظهري كما يجب<br>☐ أعتني بمظهري بشكل جيد كما كنت سابقاً | 6.أشعر بنوع من الخوف,وكأن شيئا مروعاً على وشك الحدوث:<br>A<br>☐ بالتأكيد ,وبشكل مزعج<br>☐ نعم,ولكن أقل سوءاً<br>☐ قليلا,لكنه لا يقلقني<br>☐ لا أشعر بذلك على الإطلاق |
| 7.الإحساس بضيقة الصدر دون مجهود جسدي:<br>A<br>☐ في الواقع,كثيراً جداً<br>☐ كثيراً,لا بأس به<br>☐ أشعر بذلك قليلاً<br>☐ لا أشعر بذلك على الإطلاق | 8.استطيع الضحك ورؤية الجوانتب الممتعة في الاشياء:<br>D<br>☐ كما كنت سابقاً<br>☐ أقل مما كنت سابقاً<br>☐ بالتأكيد,ليس كثيراً الآن<br>☐ لا أشعر بذل<br>☐ ك على الإطلاق |

| | |
|---|---|
| 9.أنا أتطلع للأشياء من حولي باستمتاع:<br>D<br>☐ بقدر ما يممكني فعله<br>☐ نوعا ما أقل مما اعتدت على فعله<br>☐ بالتأكيد أقل مما اعتد على فعله<br>☐ لا,على الإطلاق | 10.تأتيني دائما أفكار مقلقة:<br>A<br>☐ أغلب ألاوقات<br>☐ معظم الأوقات<br>☐ من وقت لآخر,ولكن ليس كثيراً<br>☐ أحياناً |
| 11.ينتابني إحساس مفاجئ بالهلع:<br>A<br>☐ في الواقع,في كثير من الأحيان<br>☐ غالباً<br>☐ ليس كثيراً<br>☐ لا أشعر بذلك على الإطلاق | 12.أشعر بالبهجة:<br>D<br>☐ لا,على الإطلاق<br>☐ ليس كثيراً<br>☐ في بعض الأحيان<br>☐ في أغلب الأوقات |
| 13.يمكنني الإستمتاع بقراءة كتاب جيد أو مشاهدة البرامج التلفزيونية أو الإستماع إلى الإذاعة:<br>D<br>☐ غالباً<br>☐ في بعض الأحيان<br>☐ ليس كثيراً<br>☐ نادراً جداً | 14.يمكنني الجلوس براحة والشعور بالاسترخاء<br>A<br>☐ بكل التأكيد<br>☐ عادة ما<br>☐ ليس كثيراً<br>☐ لا يمكنني ذلك على الإطلاق |
